# Nigritanine as a New Potential Antimicrobial Alkaloid for the Treatment of *Staphylococcus aureus*-Induced Infections

**DOI:** 10.3390/toxins11090511

**Published:** 2019-09-01

**Authors:** Bruno Casciaro, Andrea Calcaterra, Floriana Cappiello, Mattia Mori, Maria Rosa Loffredo, Francesca Ghirga, Maria Luisa Mangoni, Bruno Botta, Deborah Quaglio

**Affiliations:** 1 Center For Life Nano Science@Sapienza, Istituto Italiano di Tecnologia, Viale Regina Elena 291, 00161 Rome, Italy; 2 Department of Chemistry and Technology of Drugs, “Department of Excellence 2018−2022”, Sapienza University of Rome, P.le Aldo Moro 5, 00185 Rome, Italy; 3 Laboratory affiliated to Pasteur Italia-Fondazione Cenci Bolognetti, Department of Biochemical Sciences, Sapienza University of Rome, P.le Aldo Moro 5, 00185 Rome, Italy; 4 Department of Biotechnology, Chemistry and Pharmacy, “Department of Excellence 2018−2022”, University of Siena, via Aldo Moro 2, 53100 Siena, Italy

**Keywords:** natural products, alkaloids, plant secondary metabolites, β-carboline, *Staphylococcus aureus*, antimicrobial activity, cytotoxicity

## Abstract

*Staphylococcus aureus* is a major human pathogen causing a wide range of nosocomial infections including pulmonary, urinary, and skin infections. Notably, the emergence of bacterial strains resistant to conventional antibiotics has prompted researchers to find new compounds capable of killing these pathogens. Nature is undoubtedly an invaluable source of bioactive molecules characterized by an ample chemical diversity. They can act as unique platform providing new scaffolds for further chemical modifications in order to obtain compounds with optimized biological activity. A class of natural compounds with a variety of biological activities is represented by alkaloids, important secondary metabolites produced by a large number of organisms including bacteria, fungi, plants, and animals. In this work, starting from the screening of 39 alkaloids retrieved from a unique *in-house* library, we identified a heterodimer β-carboline alkaloid, nigritanine, with a potent anti-*Staphylococcus* action. Nigritanine, isolated from *Strychnos nigritana*, was characterized for its antimicrobial activity against a reference and three clinical isolates of *S. aureus.* Its potential cytotoxicity was also evaluated at short and long term against mammalian red blood cells and human keratinocytes, respectively. Nigritanine showed a remarkable antimicrobial activity (minimum inhibitory concentration of 128 µM) without being toxic in vitro to both tested cells. The analysis of the antibacterial activity related to the nigritanine scaffold furnished new insights in the structure–activity relationships (SARs) of β-carboline, confirming that dimerization improves its antibacterial activity. Taking into account these interesting results, nigritanine can be considered as a promising candidate for the development of new antimicrobial molecules for the treatment of *S. aureus*-induced infections.

This manuscript is dedicated to the memory of Professor Maurizio Botta (University of Siena, Department of Biotechnology, Chemistry and Pharmacy) who prematurely passed away on 2 August 2019. During his successfully scientific career, he synthesized a huge number of small bioactive molecules for the development of new pharmaceutical agents for cancer therapy and/or treatment of microbial infections, thus providing an invaluable contribution in the field of medicinal chemistry and drug discovery, worldwide.

## 1. Introduction

The discovery of antibiotics in the 1900s led to a medical revolution in the fight against bacterial infections. However, during the years, bacteria have developed different mechanisms to resist the killing activity of antibiotics [1]. The human pathogen *Staphylococcus aureus* is a microorganism with high adaptability and tenacity, as highlighted by its abundance in the environment and in the normal flora, the variety of virulence factors that it produces, and the capability to colonize various human organs such as nose, pharynx, and skin [2,3,4]. Furthermore, multidrug-resistant *S. aureus* is one of the major microorganisms causing bloodstream infections associated with high levels of morbidity and mortality worldwide [5]. Considering that *S. aureus* has successfully evolved numerous strategies to resist the activity of practically all antibiotics, new alternative compounds able to defeat *S. aureus*-induced infections are urgently needed [6]. Notably, a significant portion of the commercial drugs occurs in nature or is derived from natural products by means of chemical transformations or de novo synthesis [7]. Alkaloids are a group of important secondary metabolites which are produced by a wide variety of organisms including bacteria, fungi, plants, and animals. Chemically, alkaloids are a large and structurally diverse group of nitrogen-containing compounds (one or more nitrogen atoms within a heterocycle ring) [8]. Alkaloids can occur as monomers, dimers (bisalkaloids), trimers, or tetramers. According to their chemical structure, alkaloids are classified in heterocyclic alkaloids (also known as typical alkaloids), containing nitrogen in the heterocycle and originating from amino acids, and nonheterocyclic alkaloids (also known as atypical or proto-alkaloids), containing a nitrogen atom derived from an amino acid which is not a part of the heterocyclic ring [9]. Heterocyclic alkaloids are divided according to their ring structure in several classes of monomeric alkaloids (e.g., pyrrole, pyrrolidine, pyridine, piperidine, indole, quinoline, isoquinoline alkaloids). Since 1940, large-scale efforts have been made to evaluate the antibacterial effects of naturally occurring alkaloids. Several potent monomer and dimer alkaloids were identified, and synthetic modifications were investigated to improve their biological activity [8,9,10,11]. However, a tremendously wide discrepancy between their historical significance and their occurrence in modern drug development exists, and no alkaloids are available in the market as antibacterial drugs [12]. In this work, an in-house library of about 1000 natural products and their derivatives was used as a unique source of lead compounds to identify new potential antibacterial alkaloids. From the screening of all the alkaloids present in this library, the rare β-carboline heterodimer nigritanine was identified and showed a potent antistaphylococcal activity. Therefore, it was thoroughly characterized for its antimicrobial and cytotoxic activities.

## 2. Results and Discussion

### 2.1. Alkaloids Collection

Natural products remain the most productive source of leads in antibacterial drug discovery, often providing novel mechanism(s) and chemical structures as useful platforms for the development of drugs. A unique in-house library of about 1000 natural compounds, mostly isolated from several plants used in traditional medicine of South America and collected over the years, is available at the Organic Chemistry Laboratory of the Department of Chemistry and Technology of Drugs (Sapienza University of Rome, Italy). This library consists of natural products belonging to different classes of organic compounds which were previously published and fully characterized [13,14]. It was then enlarged by the addition of other natural small molecules from commercially available sources and synthetic or semi-synthetic derivatives. Currently, all components of our collection are incorporated into a virtual library, and their chemical and physicochemical features are analyzed by means of cheminformatics tools, showing a satisfactory chemical diversity. Therefore, our *in-house* library is a valid source of chemotypes for the modulation of biomolecular targets, and it was successfully screened in silico and in vitro for the identification of hit and lead compounds in previous early-stage drug discovery projects [15]. One of the largest and most intriguing classes of natural occurring compounds within the library are the alkaloids, which consist of isoquinoline (**1**–**11**), quinoline (**12**–**15**), and indole (**16**–**39**) alkaloids (Table 1). 

Considering the chemical structures of isoquinoline alkaloids, this group can be divided in two major categories: simple isoquinolines, which are composed of a benzene ring fused to a pyridine ring, and benzylisoquinolines, which contain a second aromatic ring [42]. In contrast, indole alkaloids are bicyclic structures consisting of a six-membered benzene ring fused to a five-membered nitrogen-containing pyrrole ring and are among the most numerous (at least 4100 known molecules) and complex alkaloids. In our library, the group of indole alkaloids covers several subclasses, the largest of which is represented by β-carbolines featuring a common tricyclic pyrido[3,4-b]indole ring structure (Table 1). According to the saturation of the N-containing six-membered ring, β-carbolines are categorized in fully aromatic (βCs), dihydro- (DHβCs), and tetrahydro- (THβCs) β-carbolines [43]. Alkaloids from the in-house library belonging to more than 10 plant families (e.g., Apocynaceae, Loganiaceae, Berberidaceae, Papaveraceae, Rubiaceae) are known to occur in several species (Table 1). With the aim to identify new potential antistaphylococcal agents, the in-house library of alkaloids was initially screened towards Gram(+) and Gram(-) reference bacterial strains. For the biological characterization, all the compounds were dissolved in dimethyl sulfoxide (DMSO).

### 2.2. Antimicrobial Activity

#### 2.2.1. Inhibition Zone Assay

The antimicrobial activity of all collected compounds was initially tested on a reference strain of the Gram(+) *S. aureus* (*S. aureus* ATCC 25923) by the inhibition zone assay. The Gram(-) bacterial strain *Escherichia coli* ATCC 25922 was also included for comparison (Table 2). Most of the compounds resulted to be inactive against both classes of bacteria (data not shown). The only exception was the already characterized methylated derivative of β-carboline, i.e., harmane, which was able to inhibit the growth of both *S. aureus* and *E. coli* strains in an agar diffusion assay [44,45,46], with diameters of the inhibition zone of 4.36 and 8.46 mm, respectively (Table 2).

Interestingly, a greater selectivity towards the human pathogen *S. aureus* was noted, especially for the β-carboline alkaloids. In fact, the rare heterodimer alkaloid nigritanine (compound **35**) as well as some of its analogues (i.e., speciociliatine, mytragine, and paynantheine) showed a powerful activity against the reference strain of *S. aureus* ATCC 25923, with a diameter of growth inhibition zone ranging from 8.24 to 10.39 mm. Mytragine was previously characterized for its selective anti-Gram(+) efficacy [47]; in contrast, no microbiological data have been provided so far for speciociliatine, paynantheine, and rhyncophylline. Because of these reasons, we decided to examine the activity of nigritanine, mytragine, and the other three abovementioned molecules against three multidrug-resistant clinical isolates of *S. aureus*. As reported in Table 3, nigritanine was the sole compound that retained a potent activity against the clinical isolates of *S. aureus* (i.e., *S. aureus* 1a, 1b, 1c). This was indicated by the similar diameters of inhibition zones. In contrast, all the others molecules completely or almost completely lost their activity towards *S. aureus* 1a, 1b, and 1c strains.

**Figure 1:**
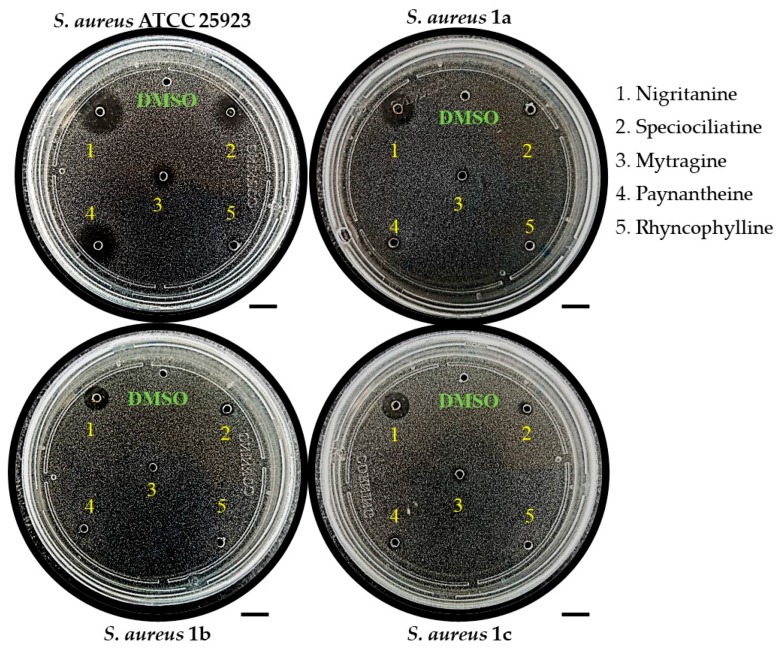
Representative image of the inhibition zone assays of nigritanine (**35**) and some other alkaloids against the reference strain and the three clinical isolates of *S. aureus*. Scale bars represent 1 cm.

A representative image of the antibacterial activity of these compounds is shown in Figure 1. The growth inhibition zone of nigritanine (zone #1) is clearly evident compared to that of the other alkaloids tested. These results are in line with other published data of alkaloids extracted from *Anabasis articulata*, showing a potent anti-Gram(+) activity when evaluated by the inhibition zone assay [48].

#### 2.2.2. Determination of the Minimum Inhibitory Concentration

The antibacterial activity of nigritanine, speciociliatine, mytragine, paynantheine, and rhyncophylline was also evaluated by the microdilution broth assay to determine the minimum inhibitory concentration (MIC) against the reference strain of *S. aureus* and the three clinical isolates after 16 hours of treatment (Table 4). Remarkably, although the MIC of nigritanine against the reference strain of *S. aureus* was higher than the MIC of other alkaloids reported in the literature (i.e., 128 μM, 56.5 μg/mL versus 2–16 μg/mL for vincamine, atropine, allantoin, or trigonelline [49]), the ability to inhibit the growth of the clinical isolates was maintained at the same concentration of 128 μM. This is in contrast with what observed for the aforementioned compounds which completely lost activity when tested against clinical isolates [49]. Similar MIC values were also obtained with alkaloids from leaves of *Eclipta alba* [50].

**Figure 2:**
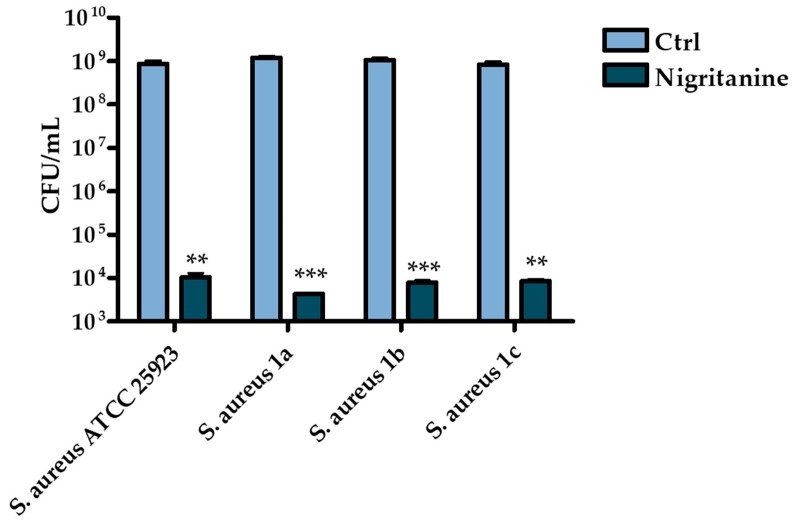
Reduction in the number of viable bacterial cells (evaluated by colony forming unit (CFU) counting) of the reference and clinical isolates of *S. aureus* strains after 16 hours treatment with nigritanine at the MIC (128 μM) compared to control (Ctrl) samples consisting in vehicle-treated bacterial cells. The data represent the mean of three independent experiments ± SD. The levels of statistical significance versus the Ctrl samples were *p* < 0.01 (**); *p* < 0.001 (***).

Notably, the MICs of nigritanine were found to correspond to the minimum bactericidal concentration (MBC) which is defined as the minimum concentration of drug causing ≥3 log killing of bacteria after 16 hours of incubation. Indeed, about five log reduction in the number of viable cells of the reference and clinical isolates of *S. aureus* were detected after treatment with nigritanine at its MIC (i.e., 128 μM) (Figure 2). Note that other plant alkaloid extracts had similar or even higher MIC and MBC values against *S. aureus* and other Gram(+) bacterial strains. For example, alkaloid extracts from the aerial part of *Sida acuta* gave MIC and MBC values ranging from 80 to >400 μg/mL against *Staphylococcus* strains [51], while the MIC of alkaloid extracts of *Mahonia aquifolium* ranged from 100 to 500 μg/mL against *Staphylococcus epidermidis* and *Staphylococcus hominis* strains [52]. Very high MIC values (>500 μg/mL) were obtained with alkaloids isolated from aerial parts of *Hypecoum erectum* L. (i.e., protopine and allocryptopine) against *S. aureus*, *Bacillus cereus*, and *Bacillus subtilis* strains [53]. Since alkaloids extracts with MICs ranging from 100 to 1000 µg/mL are considered to be compounds endowed with antimicrobial activity [54,55], nigritanine (MIC = 56.5 μg/mL) would represent a highly potential antimicrobial molecule.

### 2.3. Structure–Activity Relationships (SARs) of Nigritanine for Its Antibacterial Activity

**Figure 3:**
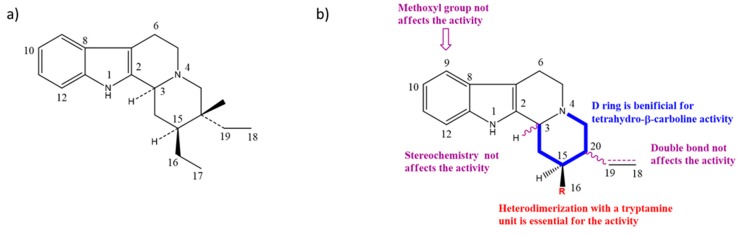
(**a**) Chemical structure of corynane. (**b**) Structure–activity relationship (SARs) analysis of tetrahydro-β-carboline alkaloids with respect to antibacterial activity.

Taking into account all these microbiological data, the new β-carboline alkaloid nigritanine (**35**) emerged as a promising antibacterial agent against *S. aureus*. Nigritanine is a rare β-carboline heterodimer from different African *Strichnos* species. In particular, the interest for the *Strichnos* genus, due to the large variety of alkaloids and their use in traditional medicine, led Nicoletti et al. to isolate compound **35**, along with other alkaloids, from the leaves of *Strichnos nigritana* Bak and from the stem bark of *Strichnos barteri* Solered, two species rather common in West Africa. From a chemical standpoint, nigritanine is a heterodimer alkaloid formed by the union of a corynane (Figure 3a) and a tryptamine unit. Interestingly, this corynane heterodimer displays a substantially higher antibacterial activity than the monomeric analogs, confirming the trend observed for the β-carboline homodimer [43,56]. The structure–activity relationships (SARs) were investigated for nigritanine (**35**) and its monomeric analogs (Figure 3b). Accordingly, the analysis of the antibacterial activity related to the corynane scaffolds indicated that: (1) the tetrahydro-β-carboline scaffold exhibits good activity; (2) the methoxyl group at C9 position, the double bond at C19–C18, and the stereochemistry of C3 and C20 do not affect the activity; (3) the corynane heterodimer shows a substantially higher activity than the monomeric analogs, highlighting that the presence of the tryptamine unit is essential. Notably, for β-carboline indoles, dimerization improves the antibacterial activity possibly because the larger molecule is less susceptible to bacterial efflux [43].

### 2.4. Cytotoxicity

#### 2.4.1. Hemolytic Assay

**Figure 4:**
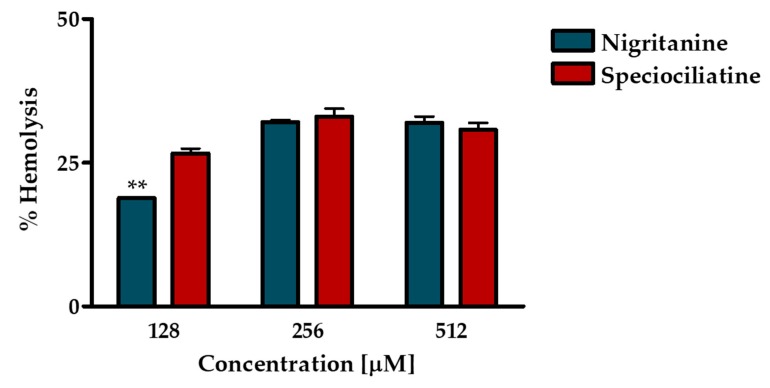
Hemolytic activity of nigritanine at 128 μM (MIC), 256 μM (2 × MIC), and 512 μM (4 × MIC) after 40 minutes of treatment compared to the least active speciociliatine. The data represent the mean ± standard error of the mean (SEM) of three independent experiments. The level of statistical significance between the two compounds was *p* < 0.01 (**).

The short-term cytotoxic effect of nigritanine was tested against mammalian red blood cells after 40 minutes treatment at its MIC (128 μM), 2 × MIC (256 μM), and 4 × MIC (512 μM). The least active compound speciociliatine was tested at the same concentrations for comparison. As reported in Figure 4, both compounds displayed a weak toxic effect, causing about 30% hemolysis at the highest concentrations, while nigritanine gave rise to about 20% lysis of erythrocytes at its active concentration (128 μM). These results confirmed the potential safety of nigritanine in mammalian cells at a short term.

#### 2.4.2. Cytotoxic Effect on HaCaT Cells

**Figure 5:**
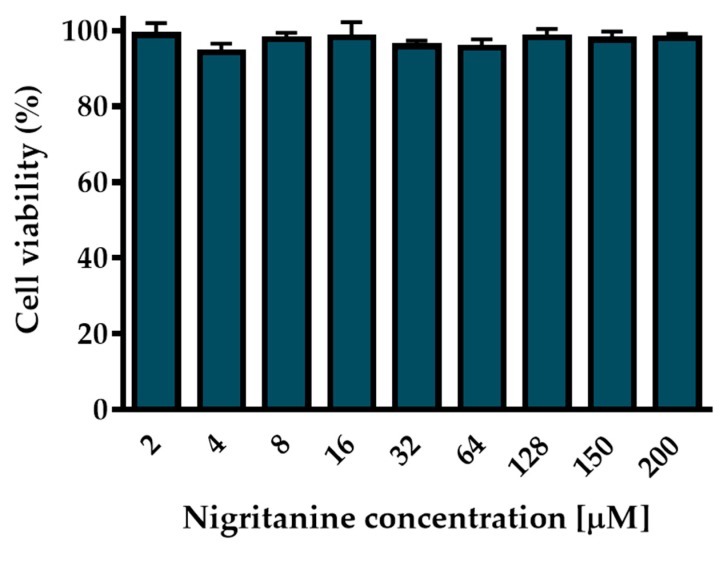
Effect of nigritanine on the viability of HaCaT cells determined by the MTT assay. Cell viability is expressed as a percentage with respect to the control. All data are the means of three independent experiments ± SEM.

Since the most common site of *S. aureus* infection is the skin, and keratinocytes represent the major cell type in the epidermis [57], the long-term cytotoxic effect of nigritanine was evaluated by the 3-(4,5-dimethylthiazol-2-yl)-2,5-diphenyltetrazolium bromide (MTT) assay on human immortalized keratinocytes (HaCaT) after 24 h treatment. As indicated in Figure 5, nigritanine did not induce any marked reduction in the percentage of viable keratinocytes after 24 h incubation at a concentration range between 2 μM and 200 μM. Note that other natural alkaloids were cytotoxic at much lower concentrations than 2 μM [49,58,59], which contrasts with the maximum non-toxic concentration tested for nigritanine (200 μM = 88.3 μg/mL). Moreover, the MICs of nigritanine (**35**) against reference and clinical isolates of *S. aureus* were equal to 128 μM. These results support the use of this compound as an antibacterial agent harmless to mammalian cells at its active antibacterial concentration.

## 3. Conclusions

With the increasing occurrence of multidrug-resistant bacterial infections and the low number of new antimicrobial agents on the market, the discovery of new natural compounds with antibiotic action is extremely necessary. In this work, we characterized the antibacterial profile of the heterodimer alkaloid nigritanine (isolated in the 1980s from *Strychnos* species), against *S. aureus* strains including clinical isolates. Interestingly, nigritanine resulted to have a potent anti-staphylococcal activity without being toxic to mammalian red blood cells and human keratinocytes at its active concentration. More importantly, it retained its antibacterial activity against three multidrug-resistant clinical isolates of *S. aureus*, a feature that was not observed for the other tested carboline alkaloids. Thus, the heterodimer alkaloid nigritanine has emerged as a promising scaffold for the design and development of potent and selective antibacterial compounds with low cytotoxicity.

## 4. Material and Methods

### 4.1. Chemistry

All the tested compounds (namely, **1**–**39**) are known structures belonging to our in-house library of natural products. The chemical identity of compounds was assessed by re-running Nuclear magnetic resonance spectroscopy (NMR) experiments and proved to be in agreement with the literature data reported below for each compound. The purity of all compounds, checked by reversed-phase High Performance Liquid Chromatography (HPLC), was always higher than 95%.

Compound **1** (dihydroberberine hydrochloride or 9,10-dimethoxy-6,8-dihydro-5*H*-[1,3]dioxolo[4,5-*g*]isoquinolino[3,2-*a*]isoquinoline hydrochloride) was purchased from Fluka (CAS: 483-15-8, St. Louis, MO, USA) and used without further purification.

Compound **2** (bulbocapnine hydrochloride or (*S*)-11-methoxy-7-methyl-6,7,7a,8-tetrahydro-5*H*-[1,3]dioxolo[4’,5’:4,5]benzo[1,2,3-*de*]benzo[*g*]quinolin-12-ol hydrochloride) was purchased from Sigma-Aldrich (CAS: 632-47-3, St. Louis, MO, USA) and used without further purification.

Compound **3** (boldine or (6a*S*)-1,10-dimethoxy-6-methyl-5,6,6a,7-tetrahydro-4*H*-dibenzo[*de,g*]quinoline-2,9-diol) was purchased from Sigma-Aldrich (CAS: 476-70-0, St. Louis, MO, USA) and used without further purification.

Compound **4** (cotarmine hydrochloride or (*R*)-4-methoxy-6-methyl-5,6,7,8-tetrahydro-[1,3]dioxolo[4,5-*g*]isoquinolin-5-ol) was purchased from MolPort (CAS: 82-54-2, Beacon, NY, USA) and used without further purification.

Compound **5** (chelidonine or (5b*R*,6*S*,12b*S*)-13-Methyl-5b,6,7,12b,13,14-hexahydro[1,3]dioxolo[4’,5’:4,5]benzo[1,2-*c*][1,3]dioxolo[4,5-*i*]phenanthridin-6-ol) was purchased from Sigma-Aldrich (CAS: 476-32-4, St. Louis, MO, USA) and used without further purification.

Compound **6** (emetine hydrochloride or (2*S*,3*R*,11b*S*)-2-(((*R*)-6,7-dimethoxy-1,2,3,4-tetrahydroisoquinolin-1-yl)methyl)-3-ethyl-9,10-dimethoxy-2,3,4,6,7,11b-hexahydro-1*H*-pyrido[2,1-*a*]isoquinoline hydrochloride) was purchased from MolPort (CAS: 14198-59-5, Beacon, NY, USA) and used without further purification.

Compound **7** ((*S*)-Glaucine or (6a*S*)-1,2,9,10-tetramethoxy-6-methyl-5,6,6a,7-tetrahydro-4*H*-dibenzo[*de,g*]quinoline) was purchased from MolPort (CAS: 475-81-0, Beacon, NY, USA) and used without further purification.

Compound **8** (hydrastine or (*R*)-6,7-dimethoxy-3-((*R*)-6-methyl-5,6,7,8-tetrahydro-[1,3]dioxolo[4,5-g]isoquinolin-5-yl)isobenzofuran-1(3*H*)-one) was purchased from Sigma-Aldrich (CAS: 118-08-1, St. Louis, Mo., USA) and used without further purification.

Compound **9** (noscapine or narcotine or (3*S*)-6,7-dimethoxy-3-((5*R*)-4-methoxy-6-methyl-5,6,7,8,9,9a-hexahydro-[1,3]dioxolo[4,5-*g*]isoquinolin-5-yl)isobenzofuran-1(3*H*)-one) was purchased from Sigma-Aldrich (CAS: 128-62-1, St. Louis, MO, USA) and used without further purification.

Compound **10** (papaverine or (6,7-dimethoxyisoquinolin-1-yl)(3,4-dimethoxyphenyl)methanone) was purchased from MolPort (CAS: 58-74-2, Beacon, NY, USA) and used without further purification.

Compound **11** (tubocurarine chloride hydrochloride or (1*S*,16*R*)-10,25-dimethoxy-15,15,30-trimethyl-7,23-dioxa-30-aza-15-azoniaheptacyclo[22.6.2.2^3,6^.1^8,12^.1^18,22^.0^27,31^.0^16,34^]hexatriaconta-3(36),4,6(35),8(34),9,11,18(33),19,21,24,26,31-dodecaene-9,21-diol chloride hydrochloride) showed NMR spectra identical to those reported in the literature [25].

Compound **12** (cinchonine or (*S*)-quinolin-4-yl((1S,2R,4S,5R)-5-vinylquinuclidin-2-yl)methanol) was purchased from Sigma-Aldrich (CAS: 118-10-5, St. Louis, MO, USA) and used without further purification.

Compound **13** (kokusaginine or 4,6,7-trimethoxyfuro[2,3-*b*]quinoline) showed NMR spectra identical to those reported in the literature [27].

Compound **14** (maculine or 9-methoxy-[1,3]dioxolo[4,5-*g*]furo[2,3-*b*]quinoline) showed NMR spectra identical to those reported in the literature [27].

Compound **15** (4-methoxy-2-(1-ethylpropyl)-quinoline) showed NMR spectra identical to those reported in the literature [27].

Compound **16** (aspidospermine or 1-((3a*R*,5a*R*,10b*R*,12b*R*)-3a-Ethyl-7-methoxy-2,3,3a,5,5a,11,12,12b-octahydro-1*H*,4*H*-6,12a-diaza-indeno[7,1-*cd*]fluoren-6-yl)-ethanone) showed NMR spectra identical to those reported in the literature [28].

Compound **17** (brucine or (4a*R*,5a*S*,8a*R*,15b*R*)-10,11-dimethoxy-4a,5,5a,7,8,13a,15,15a,15b,16-decahydro-2*H*-4,6-methanoindolo[3,2,1-*ij*]oxepino[2,3,4-*de*]pyrrolo[2,3-*h*]quinolin-14-one) was purchased from Sigma-Aldrich (CAS: 357-57-3, St. Louis, MO, USA) and used without further purification.

Compound **18** (diaboline or (4b*R*,7a*S*,8a*R*,13*R*,13a*R*,13b*R*)-13-hydroxy-5,6,7a,8,8a,11,13,13a,13b,14-decahydro-7,9-methanoindeno[1,2-*d*]oxepino[3,4-*f*]indole-14-carboxylic acid) showed NMR spectra identical to those reported in the literature [60].

Compound **19** (physostigmine or eserine or ((3a*R*,8b*S*)-3,4,8b-trimethyl-2,3a-dihydro-1*H*-pyrrolo[2,3-*b*]indol-7-yl) *N*-methylcarbamate) was purchased from MolPort (CAS: 57-47-6, Beacon, NY, USA) and used without further purification.

Compound **20** (holstiine or (15*Z*)-15-Ethylidene-10-hydroxy-17-methyl-11-oxa-8,17-diazapentacyclo[12.5.2.11,8.02,7.013,22]docosa-2,4,6-triene-9,20-dione) showed NMR spectra identical to those reported in the literature [61].

Compound **21** (pseudobrucine or (4a*R*,4a^1^*R*,5a*R*,8a*S*,8a^1^*S*,15a*S*)-5a-hydroxy-10,11-dimethoxy-4a^1^,5,5a,7,8,8a^1^,15,15a-octahydro-2*H*-4,6-methanoindolo[3,2,1-*ij*]oxepino[2,3,4-*de*]pyrrolo[2,3-*h*]quinolin-14(4a*H*)-one) was purchased from Fisherpharma (CAS: 560-30-5, Beacon, NY, USA) and used without further purification.

Compound **22** (retuline or 1-((3a*S*,5*R*,6*S*,6a*S*,11b*R*,*E*)-12-ethylidene-6-(hydroxymethyl)-1,2,4,5,6,6a-hexahydro-3,5-ethanopyrrolo[2,3-*d*]carbazol-7(3a*H*)-yl)ethanone) showed NMR spectra identical to those reported in the literature [62].

Compound **23** (serotonin or 3-(2-aminoethyl)-1H-indol-5-ol) was purchased from Sigma-Aldrich (CAS: 50-67-9, St. Louis, MO, USA) and used without further purification.

Compound **24** (triptamine hydrochloride or 2-(1*H*-indol-3-yl)ethan-1-amine hydrochloride) was purchased from Sigma-Aldrich (CAS: 343-94-2, St. Louis, MO, USA) and used without further purification.

Compound **25** (vomicine hydrochloride or (4a*R*,4a^1^*R*,6a*S*,6a^1^*S*,13a*S*)-10-hydroxy-16-methyl-4a,5,13,13a-tetrahydro-2*H*-6a,4-(ethanoiminomethano)indolo[3,2,1-*ij*]oxepino[2,3,4-*de*]quinoline-6,12(4a^1^*H*,6a^1^*H*)-dione hydrochloride) was purchased from MolPort (5969-84-6, Beacon, NY, USA) and used without further purification.

Compound **26** (vindoline or (3a*R*,3a^1^*R*,4*R*,5*S*,5a*R*,10b*R*)-methyl 4-acetoxy-3a-ethyl-5-hydroxy-8-methoxy-6-methyl-3a,3a^1^,4,5,5a,6,11,12-octahydro-1*H*-indolizino[8,1-*cd*]carbazole-5-carboxylate) was purchased from MolPort (CAS: 2182-14-1, Beacon, NY, USA) and used without further purification.

Compound **27** (akagerine or (*E*)-2-((3a*S*,5*R*,7*S*)-7-hydroxy-3-methyl-1,2,3,3a,4,5,6,7-octahydro-3,7a-diazacyclohepta[*jk*]fluoren-5-yl)but-2-enal) showed NMR spectra identical to those reported in the literature [63].

Compound **28** (canthin-6-one or 6*H*-indolo[3,2,1-*de*][1,5]naphthyridin-6-one) was purchased from MolPort (CAS: 479-43-6, Beacon, NY, USA) and used without further purification.

Compound **29** (α-carboline or 9*H*-pyrido[2,3-*b*]indole) was purchased from MolPort (CAS: 244-76-8, Beacon, NY, USA) and used without further purification.

Compound **30** (harmane or 1-methyl-9*H*-pyrido[3,4-*b*]indole) was purchased from Sigma-Aldrich (CAS: 486-84-0, St. Louis, MO, USA) and used without further purification.

Compound **31** (norharmane or 9*H*-pyrido[3,4-*b*]indole) was purchased from Sigma-Aldrich (CAS Number: 244-63-3, St. Louis, MO, USA) and used without further purification.

Compound **32** (harmine or 7-methoxy-1-methyl-9*H*-pyrido[3,4-*b*]indole) was purchased from Sigma-Aldrich (CAS: 442-51-3, St. Louis, MO, USA) and used without further purification.

Compound **33** (ibogaine or (6*R*,7*S*,11*S*)-7-ethyl-2-methoxy-6,6a,7,8,9,10,12,13-octahydro-5*H*-6,9-methanopyrido[1’,2’:1,2]azepino[4,5-*b*]indole) showed NMR spectra identical to those reported in the literature [38].

Compound **34** (mitragynine or (*E*)-methyl 2-((2*S*,3*S*,12b*S*)-3-ethyl-8-methoxy-1,2,3,4,6,7,12,12b-octahydroindolo[2,3-*a*]quinolizin-2-yl)-3-methoxyacrylate) was purchased from BOC Sciences (CAS: 4098-40-2, Shirley, NY, USA) and used without further purification.

Compound **35** (nigritanine or (2*S*,3*R*,12b*S*)-3-ethyl-2-(((*S*)-2-methyl-2,3,4,9-tetrahydro-1*H*-pyrido[3,4-*b*]indol-1-yl)methyl)-1,2,3,4,6,7,12,12b-octahydroindolo[2,3-*a*]quinolizine) showed NMR spectra identical to those reported in the literature [64]. The chemical characterization of compound **35** is reported below: Brown solid, m.p. 202-204°C; ^1^H-NMR (CDCl_3_, 400 MHz): 0.93 (t, *J* = 7 Hz, 3H, Me-C), 2.46 (s, 3H, Me-N), 3.56 (m, 1H, H-3), 6.40 (s, 1H, H-1 or H-1’), 7.80 (s, 1H, H-1 or H-1’); ^13^C-NMR (CDCl_3_, 101 MHz): 11.2 (C18), 20.7 (C6’), 21.5 (C6), 23.7 (C19), 41.9 (C20), 42.7 (N-CH_3_), 34.9 (C14), 35.0 (C16), 35.8 (C15), 51.3 (C5’), 53.0 (C5), 58.6 (C17), 59.1 (C3), 60.2 (C21), 107.1 (C7), 108.8 (C7’), 110.8 (C12), 110.9 (C12’), 117.5 (C9 or C9’), 117.7 (C9 or C9’), 118.8 (C10 or C10’), 119.3 (C10 or C10’), 120.4 (C11 or C11’), 121.4 (C11 or C11’), 126.7 (C8), 127.0 (C8’), 134.7 (C2), 135.5 (C2’), 135.6 (C13 or C13’), 135.7 (C13 or C13’); m/z (ESI+) 452 (M+, 82%), 437 (6), 408 (10), 267 (8), 253 (12), 199 (27), 185 (100).

Compound **36** (paynantheine or (*E*)-methyl 3-methoxy-2-((2*S*,3*R*,12b*S*)-8-methoxy-3-vinyl-1,2,3,4,6,7,12,12b-octahydroindolo[2,3-*a*]quinolizin-2-yl)acrylate) was purchased from BOC Sciences (CAS: 4697-66-9, Shirley, NY, USA) and used without further purification.

Compound **37** (rhynchophylline or (*E*)-methyl 2-((1’*R*,6’*R*,7’*S*,8a’*S*)-6’-ethyl-2-oxo-3’,5’,6’,7’,8’,8a’-hexahydro-2’*H*-spiro[indoline-3,1’-indolizin]-7’-yl)-3-methoxyacrylate) was purchased from BOC Sciences (CAS: 76-66-4, Shirley, NY, USA) and used without further purification.

Compound **38** (speciociliatine or (*E*)-methyl 2-((2*S*,3*S*,12b*R*)-3-ethyl-8-methoxy-1,2,3,4,6,7,12,12b-octahydroindolo[2,3-*a*]quinolizin-2-yl)-3-methoxyacrylate) was purchased from BOC Sciences (CAS: 14382-79-7, Shirley, NY, USA) and used without further purification.

Compound **39** (yohimbine hydrochloride or (1*R*,2*S*,4a*R*,13b*S*,14a*S*)-methyl 2-hydroxy-1,2,3,4,4a,5,7,8,13,13b,14,14a-dodecahydroindolo[2’,3’:3,4]pyrido[1,2-*b*]isoquinoline-1-carboxylate hydrochloride) was purchased from Sigma-Aldrich (CAS: 65-19-0, St. Louis, MO, USA) and used without further purification.

### 4.2. Microorganisms and Cell Line

The reference Gram(+) and Gram(-) strains used for the antimicrobial tests were *S. aureus* ATCC 25923 and *E. coli* ATCC 25922, respectively. The multidrug-resistant clinical isolates of *S. aureus* (1a, 1b, and 1c) were kindly provided by Professor Giammarco Raponi (Sapienza, University of Rome).

HaCaT cells (AddexBio, San Diego, CA, USA) were cultured in Dulbecco’s modified Eagle’s medium supplemented with 4 mM glutamine (DMEMg), 10% heat-inactivated fetal bovine serum (FBS), and 0.1 mg/mL of penicillin and streptomycin at 37 °C and 5% CO_2_, in 25 cm^2^ or 75 cm^2^ flasks.

### 4.3. Antimicrobial Assays

A bacterial culture inoculum was incubated at 37 °C in Luria–Bertani (LB) broth until reaching an optical density (O.D.) of 0.8 at 590 nm. For the inhibition zone assay, the bacterial culture was diluted 1:2000 and plated in LB-agarose plates. An aliquot (3 μL) of each compound at 5 mM was loaded into holes previously made in the agarose plates [65,66]. Afterwards, the plates were incubated overnight at 30 °C. Afterwards, the diameters of the inhibition zone were measured and reported in Table 2 and Table 3.

For the determination of the MICs, 50 μL of bacterial suspension (2 × 10^5^ cells/mL) in Mueller–Hinton broth (MH) was added to 50 μL MH containing serial dilutions of the compounds (from 256 μM to 2 μM) previously prepared in a 96-well plate. Controls consisted of vehicle-treated bacterial cells [67]. The plate was then incubated for 16 hours at 37 °C, and MIC was defined as the lowest concentration causing 100% visible inhibition of microbial growth. Afterwards, aliquots from the wells corresponding to the MIC of nigritanine (**35**) and to the control were withdrawn and plated onto LB agar plates for colony forming unit (CFU) counting and MBC determination.

### 4.4. Cytotoxicity Assays

To evaluate short-term cytotoxicity, selected alkaloids were tested on sheep red blood cells (OXOID, SR0051D). Aliquots of erythrocyte suspension (O.D. of 0.5 at 500 nm) in 0.9 % (*w*/*v*) NaCl were incubated for 40 min at 37 °C with three different concentrations (MIC, 2 × MIC, and 4 × MIC) of nigritanine (**35**) or the same concentrations of speciociliatine (**38**). The treated samples were then centrifuged for 5 min at 900× g. The amount of hemoglobin released in the supernatant by lysed red blood cells was measured at 415 nm using a microplate reader (Infinite M200; Tecan, Salzburg, Austria). The complete lysis was obtained by suspending erythrocytes in distilled water according to [68,69,70].

To evaluate the in vitro long-term cytotoxicity of nigritanine (**35**), a colorimetric method was employed. This assay is based on the intracellular reduction of the yellow tetrazolium salt MTT (Sigma-Aldrich, St. Luis, MO, USA) to purple formazan crystals by mitochondrial dehydrogenases of metabolically active cells. Therefore, the amount of purple color is directly proportional to the number of viable cells. About 4 × 10 ^4^ HaCaT cells resuspended in DMEMg supplemented with 2% FBS, without antibiotics, were plated in each well of a 96-well plate. After overnight incubation in a humidified atmosphere containing 5% CO_2_ at 37 °C, the medium was removed, and fresh serum-free DMEMg containing the compound at different concentration was added in each well. For controls, cells were treated with vehicle. The plate was incubated for 24 h at 37 °C and 5% CO_2_. Afterwards, the medium was discarded, and 0.5 mg/mL of MTT in Hank’s buffer (136 mM NaCl, 4.2 mM Na_2_HPO_4_, 4.4 mM KH_2_PO_4_, 5.4 mM KCl, 4.1 mM NaHCO_3_, pH 7.2, supplemented with 20 mM D-glucose) was added to each well. After 4 h incubation at 37 °C and 5% CO_2_, 100 μL of acidified isopropanol was added to each well, in order to dissolve the formazan crystals [71,72]. Absorbance was measured by a microplate reader (Infinite M200; Tecan, Salzburg, Austria) at 570 nm, and cell viability was calculated with respect to the control (cells in medium supplemented with vehicle).

### 4.5. Statistical Analysis

All data are expressed as the mean ± SD or SEM. Statistical analyses were performed using Student’s t-test with the Prism software package (GraphPad, 6.0, San Diego, CA, USA), and the differences were considered to be statistically significant for *p* < 0.05.

## Figures and Tables

**Table 1:** List of alkaloids tested in this study.

Mol.	Common Name	Chemical Structure	M.W.	Molecular Formula	Source	Ref.
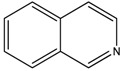 Isoquinoline Alkaloids						
**1**	Dihydroberberine·HCl	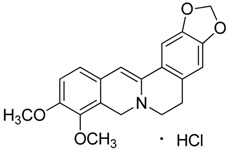	337.37 373.83 (+ HCl)	C_20_H_19_NO_4_·HCl	*Berberis* species: *Berberis aristata, Berberis lyceum, Berberis petiolaris, Berberis tinctoria* (Berberidaceae family)	[16]
**2**	Bulbocapnine·HCl	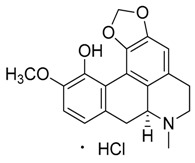	325.36 361.82 (+ HCl)	C_19_H_19_NO_4_·HCl	Species: *Corydalis cava* (Papaveraceae family)	[17]
**3**	Boldine	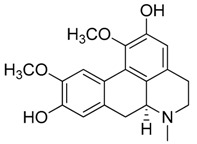	327.37	C_19_H_21_NO_4_	Species: *Peumus boldus* (Monimiaceae family)	[18]
**4**	Cotarmine·HCl	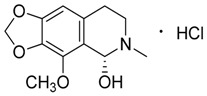	237.25 273.71	C_12_H_15_NO_4_·HCl	Synthetic	[19]
**5**	Chelidonine	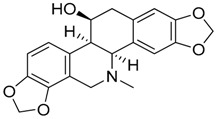	353.37	C_20_H_19_NO_5_	Species: *Chelidonium majus* L. (Papaveraceae family)	[20]
**6**	Emetine·HCl	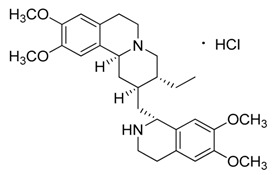	480.64 517.10 (+HCl)	C_29_H_40_N_2_O_4_	Species: *Psychotria ipecacuanha* Stokes (Rubiaceae family)	[21]
**7**	(*S*)-Glaucine	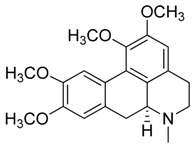	355.43	C_21_H_25_NO_4_	Species: *Glaucium luteum* L. (Papaveraceae family)	[22]
**8**	Hydrastine	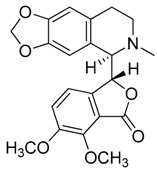	383.39	C_21_H_21_NO_6_	Species: *Hydrastis canadensis* L. (Ranunculaceae family)	[23]
**9**	Noscapine (Narcotine)	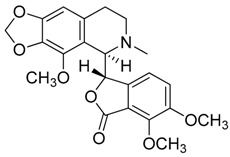	413.42	C_22_H_23_NO_7_	Species: *Papaver somniferum* (Papaveraceae family)	[24]
**10**	Papaverine	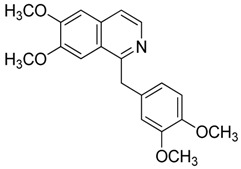	339.39	C_20_H_21_NO_4_	Species: *P. somniferum* (Papaveraceae family)	[24]
**11**	Tubocurarine Chloride·HCl	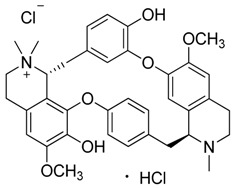	609.73 681.65 (+Cl^-^+HCl)	C_37_H_41_N_2_O_6_·HCl + Cl^-^	Species: *Liana Chondrodendron* (Menispermaceae family)	[25]
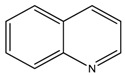 Quinoline Alkaloids						
**12**	Cinchonine	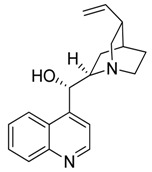	294.39	C_19_H_22_N_2_O	Species: *Cinchona ledgeriana*, *Remijia peruviana* (Rubiaceae family)	[26]
**13**	Kokusaginine	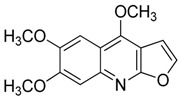	259.26	C_14_H_13_NO_4_	Species: *Esenbeckia leiocarpa* (Rutaceae family)	[27]
**14**	Maculine	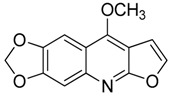	243.21	C_13_H_9_NO_4_	Species: *E. leiocarpa* (Rutaceae family)	[27]
**15**	4-methoxy-2-(1-ethylpropyl)-quinoline	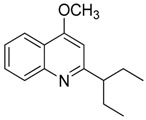	229.32	C_15_H_19_NO	Species: *E. leiocarpa* (Rutaceae family)	[27]
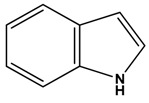 Indole Alkaloids						
**16**	Aspidospermine	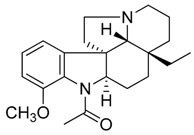	354.49	C_22_H_30_N_2_O_2_	*Aspidosperma* species: *Aspidosperma album*, *Aspidosperma australe*, *Aspidosperma exalatum*, *Aspidosperma peroba*, *Aspidosperma polyneuron*, *Aspidosperma pyricollum*, *Aspidosperma pyrifolium*, *Aspidosperma quebracho-blanco*, *Aspidosperma quirandy*, *Aspidosperma sessiflorum*, *Aspidosperma rhombeosignatum* (Apocynaceae family)	[28]
**17**	Brucine	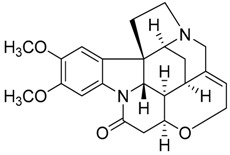	394.47	C_23_H_26_N_2_O_4_	Species: *Strychnos nux-vomica* (Apocynaceae family)	[29]
**18**	Diaboline	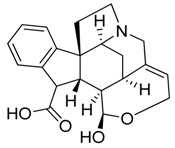	353.41	C_21_H_23_NO_4_	Species: *Strychnos castelneana* (Loganiaceae family)	[27]
**19**	Physostigmine (Eserine)	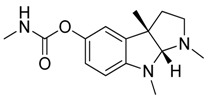	275.35	C_15_H_21_N_3_O_2_	*Physostigma venenosum* (Fabaceae family)	[30]
**20**	Holstiine	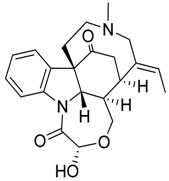	382.45	C_22_H_26_N_2_O_4_	Species: *Strychnos henningsii* Gilg (Loganiaceae family)	[31]
**21**	Pseudobrucine	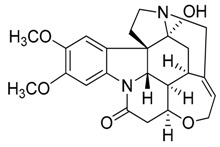	410.46	C_23_H_26_N_2_O_5_	Species: *S. nux-vomica* (Loganiaceae family)	[29]
**22**	Retuline	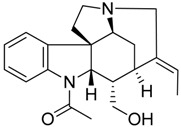	338.44	C_21_H_26_N_2_O_2_	*Strychnos* species: *Strychnos camptoneura,* *S. henningsii* (Loganiaceae family)	[31]
**23**	Serotonin	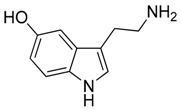	176.22	C_10_H_12_N_2_O	Species: *Laphophora williamsii* (Cactaceae family)	[32]
**24**	Triptamine·HCl	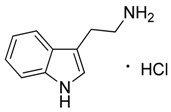	160.22 196.68 (+HCl)	C_10_H_12_N_2_ HCl	*Acacia* species (Fabacee family)	[33]
**25**	Vomicine·HC1	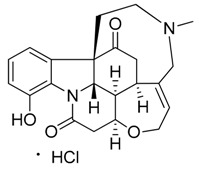	380.44 416.90 (+HCl)	C_22_H_24_N_2_O_4_ HCl	*Strychnos icaja* (Loganiaceae family)	[27,31]
**26**	Vindoline	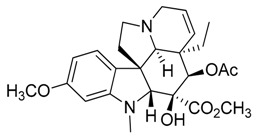	456.53	C_25_H_32_N_2_O_6_	*Catharanthus roseus* (Apocynaceae family)	[34]
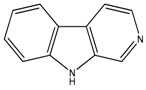 Carboline Alkaloids (Indole Subclass)						
**27**	Akagerine	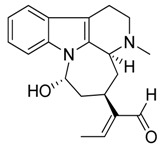	324.42	C_20_H_24_N_2_O_2_	*Strychnos* species: *Strychnos barteri* Solered, *S. camptoneurine*, *Strychnos nigritana* Bak (Loganiaceae family)	[31]
**28**	Canthin-6-one	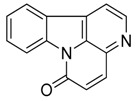	220.23	C_14_H_8_N_2_O	Species: *Simaba ferruginea* (Simaroubaceae family)	[35]
**29**	α-Carboline	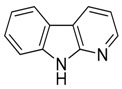	168.19	C_11_H_8_N_2_	Synthetic	[36]
**30**	Harmane	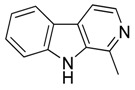	182.22	C_12_H_10_N_2_	Species: *Chimarrhis turbinata*, *Ophiorrhiza communis*, *Ophiorrhiza liukiuensis*, *Ophiorrhiza tomentosa*, *Psychotria barbiflora* (Rubiaceae family)	[26]
**31**	Norharmane	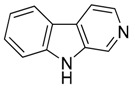	168.19	C_11_H_8_N_2_	Species: *Hygrophorus eburneus* (Tricholomataceae family)	[37]
**32**	Harmine	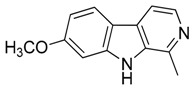	212.25	C_13_H_12_N_2_O	Species: *Banisteriopsis caapi* (Malpighiaceae family), *Grewia bicolor* (Malvaceae family), *Passiflora edulis* f. *flavicarpa* O. Deg., *Passiflora incarnata* L. (Passifloraceae family), *Tribulus terrestris* L., *Peganum harmala* L. (Zygophyllaceae family)	[37]
**33**	Ibogaine	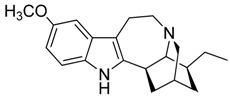	310.43	C_20_H_26_N_2_O	Species: *Tabernanthe iboga* (Apocynaceae family)	[38]
**34**	Mitragynine	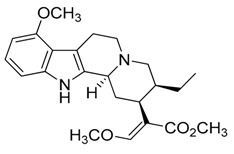	398.50	C_23_H_30_N_2_O_4_	Species: *Mitragyna speciosa* (Rubiaceae family)	[39]
**35**	Nigritanine	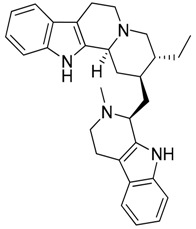	452.63	C_30_H_36_N_4_	*Strychnos* species: *Strychnos borteri*, *S. nigritana* Bak. (Loganiaceae family)	[31]
**36**	Paynantheine	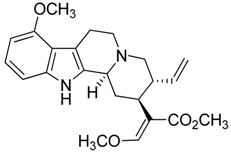	396.48	C_23_H_28_N_2_O_4_	Species: *M. speciosa* (Rubiaceae family)	[40]
**37**	Rhynchophylline	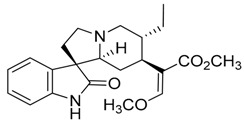	384.47	C_22_H_28_N_2_O_4_	Species: *M. speciosa*, *Uncaria rhynchophylla* (Rubiaceae family)	[40]
**38**	Speciociliatine	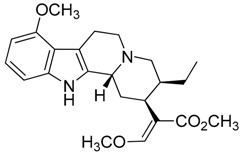	398.50	C_23_H_30_N_2_O_4_	Species: *M. speciosa* (Rubiaceae family)	[41]
**39**	Yohimbine·HCl	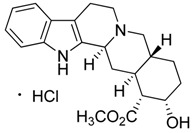	354.44 390.90 (+HCl)	C_21_H_26_N_2_O_3_ HCl	*Apocynaceae* species: *Aspidosperma discolor* A. DC., *Aspidosperma excelsum* Benth, *Aspidosperma eburneum* F. Allem, *Aspidosperma marcgravianum* Woodson, *Aspidosperma oblongum* A. DC.	[37]

Mol.: molecule number; M.W.: molecular weight; Ref.: references.

**Table 2:** Diameters of the inhibition zone of all the active tested compounds against the reference Gram(+) and Gram(-) bacterial strains.

Inhibition Zone Assay	Diameter of Inhibition Zone (mm) ^1^	
Compound	Gram-Positive *Staphylococcus aureus* ATCC 25923	Gram-Negative *Escherichia coli* ATCC 25922
Dihydroberberine·HCl (**1**)	7.800	n.a.
(*S*)-Glaucine (**7**)	7.600	n.a.
Canthin-6-one (**28**)	6.100	n.a.
Harmane (**30**)	4.360	8.640
Harmine (**32**)	n.a.	6.250
Mytragine (**34**)	5.420	n.a.
Nigritanine (**35**)	10.39	n.a.
Paynantheine (**36**)	8.440	n.a.
Speciociliatine (**38**)	8.240	n.a.

^1^ Data represent the mean of three independent experiments with standard deviation (SD) not exceeding 0.2; n.a.: not active.

**Table 3:** Diameters of the inhibition zone of some active alkaloids against the reference and clinical isolates of *S. aureus* strains.

Inhibition Zone Assay	Diameter of Inhibition Zone (mm) ^1^			
Compound	*S. aureus* ATCC 25923	*S. aureus* 1a	*S. aureus* 1b	*S. aureus* 1c
Mytragine (**34**)	5.420	4.000	n.a.	n.a.
Nigritanine (**35**)	10.39	11.20	8.440	9.100
Paynantheine (**36**)	8.440	3.800	n.a.	n.a.
Speciociliatine (**38**)	8.240	4.520	4.340	4.580

^1^ Data represent the mean of three independent experiments with SD not exceeding 0.2.

**Table 4:** Minimum inhibitory concentration (MIC) (μM) of nigritanine, speciociliatine, mytragine, paynantheine, and rhyncophylline against the reference and clinical isolates of *S. aureus* strains. MICs are the values obtained from three identical readings out of four independent experiments.

Strains	Nigritanine	Speciociliatine	Mytragine	Paynantheine	Rhyncophylline
*S. aureus* ATCC 25923	128 μM	> 256 μM	> 256 μM	> 256 μM	> 256 μM
*S. aureus* 1a	128 μM	> 256 μM	> 256 μM	> 256 μM	> 256 μM
*S. aureus* 1b	128 μM	> 256 μM	> 256 μM	> 256 μM	> 256 μM
*S. aureus* 1c	128 μM	> 256 μM	> 256 μM	> 256 μM	> 256 μM
